# Chicken Coccidiosis in Central Java, Indonesia: A Recent Update

**DOI:** 10.1155/2018/8515812

**Published:** 2018-02-08

**Authors:** Penny Humaidah Hamid, Yuli Purwandari Kristianingrum, April Hari Wardhana, Sigit Prastowo, Liliana Machado Ribeiro da Silva

**Affiliations:** ^1^Department of Parasitology, Faculty of Veterinary Medicine, Universitas Gadjah Mada, Yogyakarta, Indonesia; ^2^Department of Pathology, Faculty of Veterinary Medicine, Universitas Gadjah Mada, Yogyakarta, Indonesia; ^3^Indonesian Research Center for Veterinary Sciences, Bogor, Indonesia; ^4^Department of Animal Science, Universitas Sebelas Maret, Surakarta, Indonesia; ^5^Institute of Parasitology, Justus Liebig University Giessen, Giessen, Germany

## Abstract

Avian coccidiosis is a huge problem worldwide. Heavily infected animals that show severe clinical signs and coccidiostat resistance are causing important economic losses. The present study aimed to update the recent cases of coccidiosis in Central Java, Indonesia, and to show the importance of the disease in the region. A total of 699 samples were obtained from different chicken breed. Different* Eimeria* species were detected in 175 individuals (25.04%). Three different groups of chicken breed were considered: local chicken (autochthonous chickens of Sentul and Jawa), commercial broiler, and layer. Broiler chickens showed the highest prevalence of infection (34%), followed by layer (26.26%) and local chickens (10.45%). Mild to severe clinical signs of avian coccidiosis were observed in 42% of the infected animals, while 58% of the infected animals showed no clinical signs other than low feed conversion rates. Seven different* Eimeria *species were identified:* E. tenella *was the most prevalent (43.3%), followed by* E. maxima *(26.3%),* E. necatrix *(15.7%),* E. acervulina *(8%),* E. praecox *(3.1%),* E. mitis *(2.2%), and* E. brunetti *(1.3%). Coinfections with several* Eimeria *species were diagnosed. With this study we found massive usage of coccidiostat in the region even though its usage cannot guarantee coccidiosis-free chicken production.

## 1. Introduction

Poultry industry is the most predominant meat production in Indonesia reaching 2,030,880 tons in 2016 [1]. Nowadays, chicken meat is consumed not only as fresh meat, but also as derived products, for instance, processed food products (e.g., nugget, meatballs), frozen products, and canned meat. The increasing consumption of meat in this region is proportional to the increasing purchasing power in Asian countries observed nowadays. Further, chicken meat is cheaper than cattle or lamb meats and it is a common option to complete human demand for protein of animal origin. In fact, production of chicken increased in Asia by 68.83%, featuring the highest growth when compared to other regions such as Australia, New Zealand, Africa, and Europe [2]. In Indonesia, chicken populations are dominantly commercial broiler and layer, followed by different kinds of autochthonous/local chicken breed [3]. Different management systems are common in Indonesia, ranging from very sophisticated intensive farms to traditional production kept by small producers in villages.

Increasing production of poultry meat in Indonesia is mandatory to fulfill accelerated demands as a consequence of the increment of the population. To guarantee a high and constant production level of chicken products, diseases control programs are needed. One of the most well-known diseases worldwide is avian coccidiosis [4]. The losses caused by coccidiosis are due to not only mortality but also poor weight gain and feed conversion rate together with the costs of treatment [5]. Avian coccidiosis is caused by* Eimeria *species, which are highly species-specific. Each of the seven species known to infect chicken has a special predilection site in the intestine to complete its life cycle [6]. General clinical signs, being reduced weight gains and egg yields, together with anorexia and poor feed utilization, pale combs, and dehydration, associated with mucus and/or bloody diarrhoea, are common. Besides, coccidiosis is a predisposing factor for necrotic enteritis, whose incidence has been rising due to the stop of antibiotic usage in some countries [7, 8]. Mechanism of how* Eimeria* promote* Clostridium* spp. massive growth is not exactly understood. As reviewed elsewhere [7], several factors contribute to clostridial pathogenicity in intestine of* Eimeria* infected chicken. Coccidiosis led to chicken immunodepression, with consequent increased proliferation, adhesion, and toxin production of* Clostridium *spp. [7]. There are seven species causing chicken coccidiosis:* E. tenella *(haemorrhagic typhlitis) and* E. necatrix *(haemorrhage of the small intestine) considered highly pathogenic,* E. brunetti *(necrotising enteritis) that is slightly less pathogenic,* E. maxima* and* E. acervulina *that cause mild to severe catarrhalic enteritis, and* E. mitis* and* E. praecox *considered as low pathogenic species [9]. In fact, previous experiments using* E. praecox *evince different virulence between strains and even exceed* E. acervulina* which is known as more pathogenic species [10]. The phenomenon enhances variability of clinical manifestation in chicken, which is influenced by total dose ingested, strains virulence, and also susceptibility of the host when infection occurred [10, 11].* Eimeria* spp. life cycle is direct with short prepatency periods of 4–6 days and, consequently, the disease spreads fast between hosts. Shortly after ingestion of sporulated oocysts, invading sporozoites penetrate epithelial intestinal cells and endogenous development proceeds with 2–4 merogonies and gamogony. Observations of the animals as well as necropsies to determine the location and type of the lesions (lesion scores) in each flock are important to confirm diagnosis and identify the species virulence upon outbreak.

It has been known that avian coccidiosis is a serious threat in Indonesian poultry industry. Infections by* Eimeria* spp. in chickens reared in traditional farming management system reached 39.3% [12] with* E. tenella* as the most prevalent species. Technical services of farm industries of commercial layer and broiler chickens reported cases from many areas in Java, Kalimantan, and Sumatra islands. Nowadays and contrary to what is expected, coccidiosis cases are occurring not only in traditional management systems where no treatments or control measures are being performed, but also in intensive and semi-intensive farms where treatments and control measures are considered. For example, chemoprophylaxis with anticoccidials in feed or drinking water is performed in broilers throughout the whole fattening period until premarketing withdrawal and in laying hens until the start of egg laying to avoid residues of anticoccidial drugs in the eggs [6]. Furthermore,* Eimeria* spp. resistance to several anticoccidial drugs has been reported [13], with the emergence of drug-resistant strains of all* Eimeria *species in chickens [6]. Additionally, persistent* Eimeria* spp. infections in Indonesia may also be supported by the high environmental humidity which might contribute significantly to the completion of the parasite life cycle [14]. Given that the oocysts remain in the environment after being shed [15] and that wet floors favour oocysts sporulation, infection is maintained.

In this study, we provide recent information of coccidiosis cases in Central Java, Indonesia. The update of chicken coccidiosis situation is pivotal for consideration of control strategies as both preventive and cure programs in the area, in order to fulfil the high demands for chicken products.

## 2. Materials and Methods

Ethical clearance regarding the experiment was issued by ethics committee, LPPT Gadjah Mada University, number 00087/04/LPPT/VII/2017.

Random sampling was performed in flocks of traditional and semi-intensive management systems allocated in Central Java and Special Region of Yogyakarta in the following provinces: Kulon Progo, Gunung Kidul, Bantul, Yogyakarta, Magelang, and Boyolali, 7°47.44 south latitude and 110°8.24 eastern longitude ordinates.

**Figure 1:**
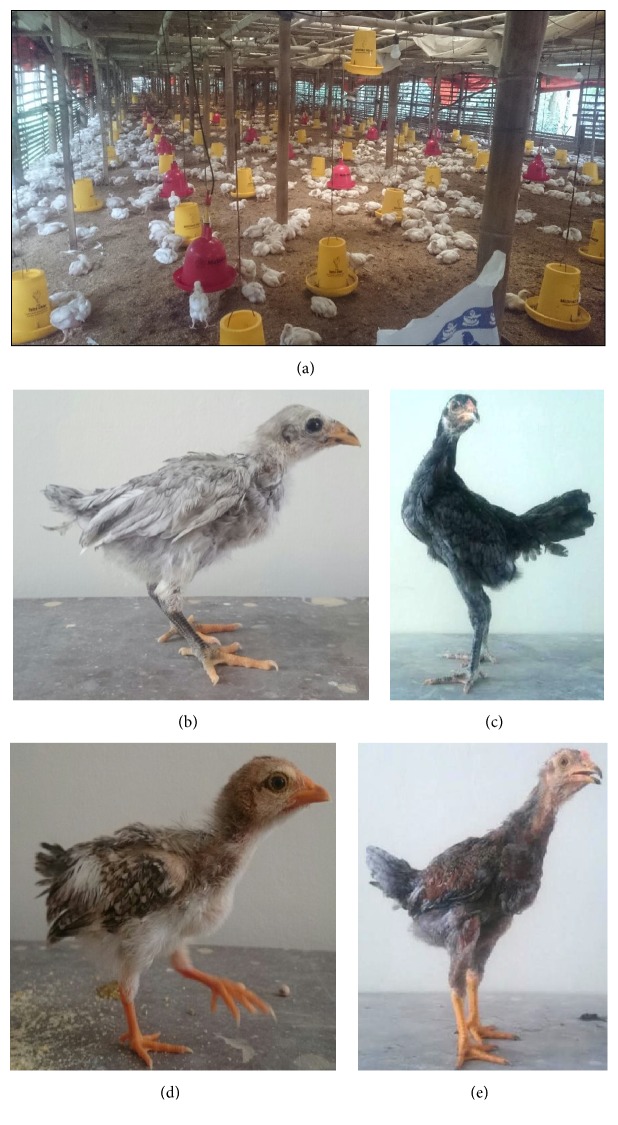
Conditions of flocks with coccidiosis in a semi-intensive farming system in Central Java. (a) Semi-intensive farming of broilers (19 days old), with almost all chicken positive for* E. tenella *infection, showing unspecific clinical signs. Sentul breed in (b) 5-day-old and (c) 21-day-old cases. Jawa breed in (d) 5-day-old and (e) 21-day-old cases.

In total 47 flocks were visited. Sample collection was carried out from April to June 2017. Chicken breeds included in this study were autochthonous chickens from Sentul and Jawa breed, commercial layers, and broilers. The Jawa chicken (Figures 1(d) and 1(e)) is characterized by mottled feather almost in whole body part and yellow skin and beaks. While Sentul chicken (Figures 1(b) and 1(c)) has more homogeneous grey color, its skin and beaks are also grey-colored and the color becomes darker in older chicken. Sentul chicken has an average of adult body weight in male of 2600 ± 207 g and female of 1408 ± 123 g; this chicken is able to produce 17 ± 1 eggs per laying period. Jawa chicken, commonly known as “ayam kampung” chicken, has an adult body weight in males of 1600 ± 107 g, females 1450 ± 150 g, and it is able to produce 13 ± 2 eggs per laying period. Jawa and Sentul chicken are farmed for meat production. These two chicken breeds are different from Bekisar (hybrid from jungle fowl) which is usually reared by citizens in Java Island due to its fascinating sound and mostly for contest or hobby purposes. It is difficult to obtain Bekisar fertile eggs, so certain breeders are producing fertile eggs by traditional mating of* Gallus varius *(as parent stock), which is now increasingly rare except in certain island, and or artificial insemination with female of Jawa chicken.

Faecal samples from chickens presenting severe or mild clinical signs of coccidiosis, as well as from animals without clinical signs, were randomly collected. Flotation technique with saturated NaCl [16] was performed to determine the presence of* Eimeria *spp. oocysts. Faecal samples containing oocysts were mixed with potassium dichromate (2% final concentration) and allowed to sporulate at room temperature.* Eimeria *species present in each sample were identified according to the morphology of the sporulated oocysts. Moreover, some chickens presenting severe clinical signs of coccidiosis, such as bloody diarrhea, were collected for further investigations at the laboratory. Necropsy of chickens with severe clinical signs was performed and lesion score was determined. Additionally, histopathology sections were performed and analysed after haematoxylin and eosin staining.

## 3. Results and Discussion

Of the total 699 samples obtained from different chicken breed,* Eimeria* spp. oocysts were detected in 175 of the chicken (25.04%). Broiler chicken showed the highest prevalence with 34% of the animals infected, followed by layer (26.26%) and local chicken (10.45%) as presented in Table 1. Clinical signs were observed in 42% of the infected chicken ranging from mild (lethargy and light diarrhoea) to severe clinical signs such as bloody diarrhoea. Nonetheless, 58% of the infected animals (positive for presence of oocysts by flotation technique) did not present any clinical signs usually described for coccidiosis. Local chicken of Sentul and Jawa (Figure 1) mostly from villages showed less prevalence of* Eimeria* spp. oocysts (Table 1).

**Figure 2:**
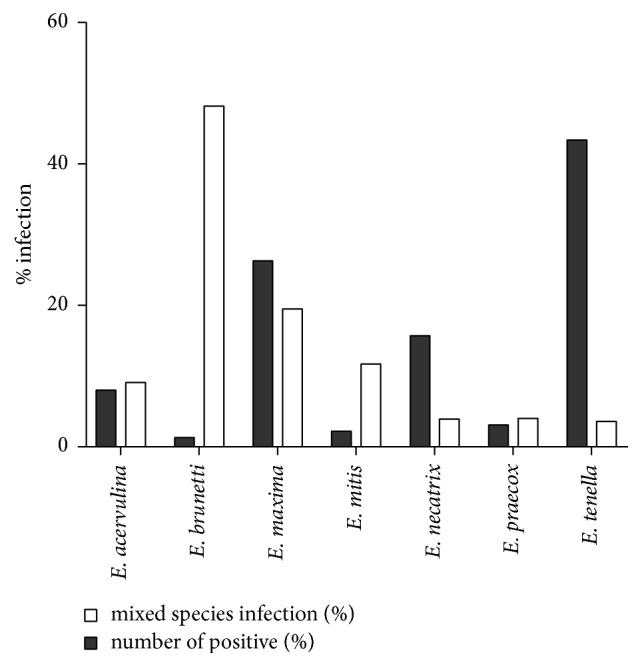
*Eimeria* species identification in positive faecal samples.

Seven* Eimeria *species were identified during this study.* E. tenella *was the most prevalent species with 43.3% prevalence, followed by* E. maxima* with 26.3%,* E. necatrix* with 15.7%,* E. acervulina* with 8%,* E. praecox* with 3.1%,* E. mitis* with 2.2%, and* E. brunetti* with 1.3% (Figure 2). Several* Eimeria *spp. with less pathogenic characteristics were found in coinfection (Figure 2). All these seven* Eimeria* spp. are evidently spread worldwide [17].* Eimeria* species have been found with different prevalence during regional surveys in France [18], Jordan [19], Romania [20], Brazil [21], or China [22]. Our findings are in agreement with previous reports that show* E. tenella *as the predominant species found in Asia [23, 24].

**Figure 3:**
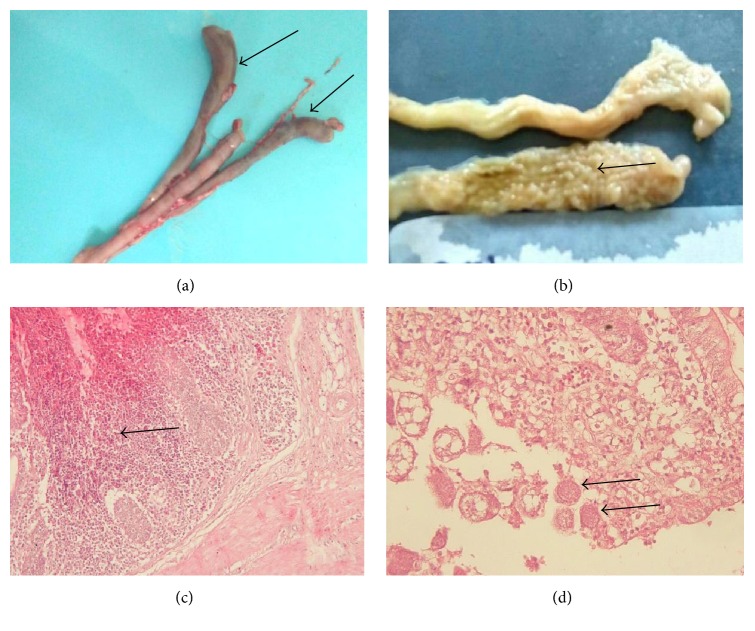
Macroscopic lesions and histopathologic sections of chicken intestine with coccidiosis in Central Java. (a) Macroscopic lesion of the caecum showing ballooning condition (arrows) with bloody lumen. (b) Different pinhead size structures were massively formed in lumen of caecum (arrow). (c) Haemorrhagic and inflammatory cells infiltration (arrow) in the infected caecum. (d) Macrogamonts of* Eimeria* spp. in the caecum (arrows). The species is identified as* E. tenella* based on the predilection site (caecum), morphology of oocyst, and sporulation time of collected oocysts from faeces.

Animals infected with less pathogenic* Eimeria* species,* E. maxima*,* E. acervulina*,* E. praecox*, and* E. miti*s, showed no clinical signs.* Eimeria tenella *was the most frequent species found in this study in comparison to other pathogenic* Eimeria*, that is,* E. necatrix* and* E. brunetti*. Although considered to be the most pathogenic species, clinical symptoms of some chicken infected with* E. tenella* were not quite clear. Mortality was never observed massively in a short period of time but it consistently occurred during fattening periods. It was observed that chicken with slight bloody diarrhoea still presented normal appetite and food intake although body weight conversion was diminished when compared to broiler chicken at the same age. Pathologic findings such as caeca ballooning were only observed when chickens were necropsied. Necropsied chickens infected with* E. tenella* showed clinical manifestation of bloody diarrhoea and ballooning caeca with various parasitic stages (Figure 3). Some chicken showed grooves in caeca lumen (Figure 3) categorized as having score 2 in* E. tenella* infection. Score lesions could not be identified in all necropsied chicken in accordance with species-specific pathogenicity since mixed infections were commonly observed. Intestines parts with no bloody diarrhoea did not show erythrocytes extravasations out of blood vessels but massive lengthened villi were observed in gross section (Figure 3).

This study also showed that coccidiosis affects all chicken examined: layer, broiler, and local chicken, Sentul and Jawa. Nevertheless, local chickens had less prevalence in comparison with broiler and layer. We collected faecal samples from chicken reared free in the villages where they have enough space for exercises and more natural feed supply. In contrast to broiler and layer examined, farmers usually use coccidiostat in feed during layer and growing/fattening period of local chicken. However, no evidence existed so far in resistance and susceptibility of these local chickens to avian coccidiosis. These current data exhibited local chicken performance in facing field infection and may show ability in eliminating infection naturally. That resistance to avian coccidiosis is inherited and genes-associated was reported elsewhere [25, 26]. So far, reports in Indonesia related to parasite-resistance breed was checked in thin-tail sheep which is less susceptible to* Fasciola hepatica* and* F. gigantica* infections [27]. In this context, further study of resistance to avian coccidiosis in local chicken breed will be useful and informative for breeding selection. Local chickens in this study (Jawa and Sentul) had lower body weight at the same age compared to broiler. However, some people choose to consume meat from local chicken due to the taste preference, therefore having higher price. Price per kg body weight of a local chicken cannot be compared to broiler; thus it is the reason why some farmers prefer to grow local chicken than broiler although its growth rate is slower. Additional information of coccidiosis-resistant phenotype in this study will benefit local chicken farming and may give insight into breeding strategy of local chicken breeds.

In the flocks of broiler sampled in this study, coccidiostat was supplemented in the feed and applied during almost the whole life of chicken. Exemplary of this observation, all chickens in Figure 1 were randomly sampled and all presented ballooning caeca although coccidiostat as feed additive was being applied. The flock of Figure 1 consisted of 5,000 chickens with semi-intensive farming system. This incidence may reflect a case of coccidiostat resistance in the field. Nowadays, avian eimeriosis resistance to various commercial anticoccidial drugs is reported and reviewed worldwide [22, 28–30]. This might be a concern since no scientific reports are published in Indonesia so far. Combination or rotation of coccidiostat has to be performed in order to prolong drug efficacies and prevent resistance to specific active compound. Instead of chemical compounds, natural feed additives can be considered to enhance chicken performance during critical phases of fattening period. Research in natural herbs shows evident improvement of immune response to avian* Eimeria* infections [29, 31–33]. Moreover, combination of live attenuated vaccines with anticoccidial drugs may enhance immune system for infection. This approach is considered effective in improving chicken endurance in accordance with application of coccidiostats [34]. In addition, a wise decision in choosing vaccination strategy is mandatory through update of information and vaccine products in the market with all its pros and cons [34].

Several farms with infected chicken showed clearly that litter management may contribute to parasite persistence and hard elimination. The humidity of litter made from rice husk (Figure 1) was observed to be high. Moisture of litter together with its surface temperature in Indonesia tropical climate is presumed to be very close to the optimum conditions for sporulation, as reviewed elsewhere [34]. However, total oocyst counts in the litter need to be performed to evaluate contribution of the litter in* Eimeria* persistency within a flock, but that was not performed in this study. Some observed farms for broiler were also having very short period of transition times before entering new brooding period of broiler for new rearing batch giving a very limited time to clean and rest the parasites cycles. Overall results in this study imply evaluation of management systems of flocks, including litter, in-out processes, vaccination, and/or combined vaccine-coccidiostat application during fattening.

## 4. Conclusion

Chicken coccidiosis is a persistent problem in Central Java. Most of the animals were asymptomatic but showing low feed conversion rates. In addition, local chicken breed, that is, Sentul and Jawa, showed less prevalence than broiler and layer. This study also pointed out that massive usage of coccidiostat in feed cannot guarantee chicken free from coccidiosis.

## Figures and Tables

**Table 1:** Prevalence of chicken coccidiosis in Central Java by chicken type.

	Chicken type		
	Broiler	Layer	Local
Total number of samples	300	198	201
Number of positive samples	102	52	21
Number of negative samples	198	146	180
Prevalence of chicken coccidiosis (%)	34.00	26.26	10.45
